# Evaluation of the features of cystic echinococcosis with concurrent super-infection: a retrospective study in Southern Iran

**DOI:** 10.1186/s12879-023-08520-5

**Published:** 2023-08-10

**Authors:** Reza Shahriarirad, Ramin Shekouhi, Amirhossein Erfani, Mohammad Rastegarian, Mehrdad Eskandarisani, Marjan Motamedi, Bahador Sarkari

**Affiliations:** 1 Thoracic and Vascular Surgery Research Center, Shiraz University of Medical Sciences, Shiraz, Iran; 2 Student Research Committee, Shiraz University of Medical Sciences, Shiraz, Iran; 3 Colorectal Research Center, Shiraz University of Medical Sciences, Shiraz, Iran; 4 Department of Parasitology and Mycology, School of Medicine, Shiraz University of Medical Sciences, Shiraz, Iran; 5 Basic Sciences in Infectious Diseases Research Center, Shiraz University of Medical Science, Shiraz, Iran

**Keywords:** Cystic echinococcosis, Fungal superinfection, Hydatid cyst, Superinfection

## Abstract

**Objective:**

Superinfection of cystic echinococcosis (CE) is a life-threatening complication with significant morbidities, which can be prevented with early diagnosis and treatment. This study aims to examine the clinical characteristics, diagnostic methods, and treatment options for superinfected CE, as there is currently limited information available on the differences between superinfected and non-infected CE in terms of clinical features, serological and radiologic findings.

**Methods:**

This cross-sectional study was conducted on hospital records of patients who were diagnosed with hydatid cysts in a 15-year period (2004 to 2018) in two main university-affiliated referral centers in Fars province, southern Iran. Patients’ information regarding the demographical and clinical features related to CE, age, sex, previous history of CE or recurrence, size and location of CE, and length of hospital stay were collected. Moreover, the characteristics of concurrent infections with hydatid cysts were recorded.

**Results:**

A total of 586 surgeries due to CE were performed on 501 patients, of which 67 (11.43%) had reoperations due to the recurrence of the disease. A total of 30 (5.99%) incidences of superinfection were observed. There were no statistically significant differences in terms of laboratory and imaging findings between CE patients with concurrent infections and other CE patients (p-value > 0.05). Among the patients with super-infection, four had fungal infections of the lungs. *Aspergillus fumigatus* was the causative pathogen in all four patients that were diagnosed with fungal superinfection. All patients underwent surgical excision with favorable long-term outcomes.

**Conclusion:**

Our study revealed a 5.99% incident rate of CE superinfection. Regarding the concurrent fungal infections in hydatid cysts, the patient’s symptoms and laboratory and imaging findings are inconclusive and histopathological evaluation seems to be the most reliable option. Surgical resection is the gold-standard treatment option with favorable outcomes and potentially can be curative.

## Introduction

Cystic echinococcosis (CE), caused by *Echinococcus granulosus*, is a neglected tropical disease that is responsible for considerable morbidity and mortality in affected patients [1]. The life cycle of *Echinococcus granulosus* occurs when definite hosts, which are mainly canids, release the eggs through feces. After ingestion by the intermediate hosts (sheep, goats, swine, and other herbivores), the eggs will hatch into oncospheres and spread through major organs. The cycle continues on after ingestion of the cyst-containing organs of the intermediate hosts [2]. Humans are the dead-end intermediate hosts that become infected after ingestion of any egg-contaminated food sources [3]. Subsequent spreading of the oncospheres via blood or lymphatic system to the liver, lungs, brain, and other major organs, causes the systematic presentation of this disorder. The clinical presentations of CE vary widely based on their primary location, size, and concurrent co/super-infection. Initially, CE remains clinically silent; however, as the disease progresses, the risks of complications such as rupture, calcification, and concurrent cystic infections increase [4].

Cyst rupture is one of the most common complications associated with CE, which is usually caused spontaneously or due to trauma. CE rupture is most often presented with acute abdominal pain with peritoneal signs that may trigger a life-threatening anaphylactic reaction caused by activation of the alternative complement pathway [5]. Superinfection is another common complication of CE that can further predispose them to rupture. Superinfection of CE occurs mostly due to either loss of membrane integrity of the cysts and subsequent communication with the adjacent structures (such as the biliary or bronchial tree) or bacteremia [6]. Although having a previous biliary or bronchial disease is a major risk factor that can increase the risk of infected CE, the majority of patients with superinfected hydatid cysts do not have such disorders. It is thought that as the cysts enlarge, they distort the adjacent bronchial and biliary trees due to mass effect, which can lead to CE superinfection [7]. Bacterial superinfections, especially Gram-positive cocci (i.e., *Staphylococcus aureus*, *Streptococcus viridans*, *Enterococcus* species) and Gram-negative bacilli (i.e., *Escherichia coli*, *Acinetobacter*), are the most common bacteria isolated from the infected hydatid cysts [8].

Fungal superinfection of a primary hydatid cyst has been very rarely reported in the literature and almost always occurs in pulmonary CE [9]. One of the most robust data came from the study by García et al. [7]. They investigated 37 cases of infected hydatid cysts with only 3 (8.1%) patients that were diagnosed with superinfected pulmonary CE by *Aspergillus fumigatus* [7]. *Aspergillus fumigatus* is the most common subspecies of *Aspergillus* that can cause invasive/noninvasive aspergillosis, aspergilloma, and allergic pulmonary aspergillosis in humans. The coexistence of CE and *Aspergillus* is an extremely rare entity and it has been only reported in a few case reports and small case series [10–12]. There is a lack of data regarding the clinical symptoms, diagnosis, management, and long-term outcome of fungal superinfection of hydatid cysts in the literature, and most studies reported till now consist of case reports. This cross-sectional study investigated the coexistence of hydatid cysts with both bacterial and fungal superinfections.

## Methods

In this cross-sectional retrospective study, data were retrieved for a 15-year period (2004 to 2018) from two main university-affiliated referral centers (Nemazi and Shahid Faghihi Hospital), located in Fars, Southern Iran. Cases were included only based on a definite final diagnosis of any type of CE at hospital discharge. CE was diagnosed through histopathological evaluation and confirmation, or clinical and radiological features in favor of CE along with positive serological test (mainly Counter Current Immunoelectrophoresis). Hospital records were extracted based on the unique disease code for the international classification of diseases (ICD), which included 122.9, and 122.8 for ICD9 and B67.8, K77.0, B67.9, and J99.8 for ICD10. Suspected cases were not included in the study. The medical records were searched manually by members of the research team which consisted of physicians as well as medical students. It is worth mentioning that a comprehensive report of the cases in our center based on our data collection has been previously reported [3].

We evaluated all hospital records whether they fulfilled the criteria for super infection based on their documented hospital records. Superinfection was assessed based on preoperative and intraoperative findings. Based on the preoperative management, if the patient had high ESR or CRP levels, or complained of sour flavor in their saliva, fever, chills, productive cough, change in the color of sputum, we suspected a ruptured hydatid cyst. Based on radiological evaluation, intact cysts without air or crescent signs mostly suspect unruptured cyst without superinfection, however, in the presence of a dropped water lily sign we suspect an infected hydatid cyst. The most definite method for evaluation of superinfection in our study was based on operation note findings, in which in the context of cyst during suction is turbid or contains frank puss [7, 13]. Periodic acid Schiff or Grocott’s methenamine silver were used to find out in tissue biopsies suspicious of *Aspergillus* co-infection [7].

Patients’ information regarding the demographical and clinical features related to CE, age, sex, previous history of CE or recurrence, size and location of the cyst, and length of hospital stay were collected and entered into SPSS version 26.0.

### Statistical analysis

Data were categorized and analyzed in aspects, one regarding patients’ features, without considering the recurrence of disease, and the other based on hydatid cyst-related features among all cases of surgery. The normality of data was evaluated with the Kolmogorov-Smirnov test. Data were reported as frequency and percentage (%), mean and standard deviation (SD), or median and quartiles. For data analysis, the Chi-square and Fisher’s exact test was used for categorical variables, while the independent sample t-test and Mann-Whitney U test for continuous variables. A P-value of less than 0.05 was considered statistically significant.

## Results

A total of 586 surgeries due to CE were performed during the 15 years from 2004 to 2018. These operations were performed among 501 patients, of which 67 had reoperations due to the recurrence of the disease. Of all 586 cases, 34 (5.80%) had a primary super-infection, of which 4 (11.7%) were during the recurrence of the disease. Therefore, a total of 30 (5.99%) incidences of superinfection were observed among the 501 patients with a diagnosis of CE. Table 1 demonstrates the features among patients with super-infection in our study and its comparison to the total features of hydatid cysts undergoing surgery due to CE.

**Table 1:** Features of patients undergoing surgery due to hydatid cyst with concurrent super-infection in Southern Iran from 2004–2018

Patient-related features		Superinfection		P-value*
		**Positive**; *n = 30*	**Negative**; *n = 471*	
**Age** (years); mean ± SD		43.8 ± 17.5	34.4 ± 19.9	0.011
**Age group** (years); n (%)	< 10	0 (0)		0.03004
	10–19	1 (3.3)	69 (14.8)	
	20–29	4 (13.3)		
	30–39	10 (33.3)	84 (18.0)	
	40–49	5 (16.7)	50 (10.7)	
	≥ 50	10 (33.3)		
**Gender**; n (%)	Male	11 (36.7)		0.17816
	Female	19 (63.3)		

* Independent sample t-test or Chi-square test.

As demonstrated in Table 1, there was a significant association between age and the occurrence of super-infection, in which patients with super-infection had higher average age, while also being observed in older age groups. The features of CE operated in our center were evaluated (N = 586), and compared based on the occurrence of super-infection (Table 2). There was no significant association between the location of the cyst with the occurrence of superinfection. Three of the cases with superinfection had simultaneous liver and lung CE, however, only in one of the cases both cysts were infected. There were no reports of infected cysts in locations other than the liver and the lung. Patients with superinfection had a significantly higher hospital stay (P = 0.005).

**Table 2:** Evaluation of hydatid cyst features with concurrent super-infection among surgical cases in Southern Iran from 2004–2018

Hydatid-Cyst related features		Superinfection		P-value*
		**Positive**; *n = 34*	**Negative**; *n = 586*	
**Location**; n (%)	Lung	21 (61.8)	258 (46.7)	0.089
	Liver	16 (47.1)	323 (58.5)	0.189
	Both	1 (2.9) ^a^	55 (9.4)	0.057
**Number of Cysts**; n (%)	1	29 (6.3)	298 (93.7)	0.574
	2	4 (3.8)	101 (96.2)	
	≥ 3	5 (7.1)	65 (92.9)	
**Serological detection**; n (%)		4 (6.7)	56 (93.3)	0.567
**Surgical Method**; n (%)	Radical	14 (8.8)	146 (91.3)	0.063
	Conservative	20 (4.7)	404 (95.3)	0.063
**Laboratory Data**; median [Q1 – Q3]	WBC (x10^9^/ml)	8.1 [6.4–11.3]	9.9 [7.1–13.1]	0.070
	Neutrophile (%)	75.8 [58.4–86.4]	76.8 [55.9–86.5]	0.945
	Lymphocyte (%)	16.0 [11.2–22.4]	13.6 [7.3–27.7]	0.448
	Eosinophile (%)	5.0 [1.0–5.0]	6.7 [3.0–12.0]	0.644
	Hemoglobin (g/dL)	11.9 [10.2–12.9]	12.1 [11.0–13.6]	0.104
	Platelet (10^3^/ml)	283.0 [205.8–391.3]	263.5 [203.3–357.8]	0.430
**Duration of hospital stay**; median [Q1 – Q3]		12.0 [7.0–17.8]	7.0 [5.0–12.0]	**0.005**
***Lung Hydatid Cyst Features***				
**Laterality**; n (%)	Right lung	11 (8.3)	121 (91.7)	0.950
	Left lung	8 (7.3)	101 (92.7)	
	Bilateral	1 (9.1)	10 (90.9)	
**Location**; n (%)	Right Upper	1 (3.7)	26 (96.3)	0.700
	Right Middle	2 (6.7)	28 (93.3)	1.000
	Right Lower	5 (6.8)	69 (93.2)	0.784
	Left Upper	4 (8.7)	42 (91.3)	0.983
	Left Lower	5 (7.9)	58 (92.1)	0.848
**Largest diameter (cm)**; median [Q1 – Q3]		5.0 [4.0–6.25]	7.0 [5.0–10.0]	0.080
**Area (cm**^**3**^**)**; median [Q1 – Q3]		19.6 [14.1–28.3]	27.5 [15.7–51.4]	0.370
**Calcification**; n (%)		2 (13.3)	13 (86.7)	0.214
**Ruptured**; n (%)		1 (4.5)	21 (95.5)	1.000
**Multiloculated**; n (%)		2 (33.3)	4 (66.7)	0.042
**Necrosis**; n (%)		0 (0)	2 (100)	1.000
**Radiological detection**; n (%)	CT	15 (9.7)	140 (90.3)	1.000
	Sonography	3 (5.6)	51 (94.4)	0.533
	X-ray	3 (8.6)	32 (91.4)	0.510
***Liver Hydatid Cyst Features***				
**Laterality**; n (%)	Left	6 (6.5)	87 (93.5)	0.770
	Right	11 (5.0)	209 (95.0)	0.520
**Largest diameter (cm)**; median [Q1 – Q3]		8.0 [6.0–11.0]	9.50 [4.3–10.8]	0.877
**Area (cm**^**3**^**)**; median [Q1 – Q3]		44.45 [14.3–84.4]	44.0 [23.6–78.5]	0.806
**Calcification**; n (%)		3 (7.3)	38 (92.7)	0.724
**Ruptured**; n (%)		0 (0)	9 (100)	1.000
**Multiloculated**; n (%)		2 (7.7)	24 (92.3)	0.658
**Radiological detection**; n (%)	CT	11 (7.0)	147 (93.0)	1.000
	Sonography	7 (3.3)	205 (96.7)	1.000
	X-ray	0 (0)	8 (100)	0.200

^a^ Among three cases with simultaneous lung and liver disease in the infected group, only one had a concurrent infection in both the lung and liver

* Chi-Square or Fisher’s exact test for categorical variables and Mann Whitney U test for continuous variables.

Bold values indicate a significant association

CT: Computed tomography; WBC: White blood cell

The most frequent medications administered for patients with superinfection were third-generation cephalosporins (Ceftriaxone), which was prescribed for 26 (76.5%) cases. Furthermore, Lincosamides (Clindamycin) were prescribed for 19 (55.9%) patients, anthelmintic (Albendazole) for 15 (44.1%) patients, Metronidzaole for 15 (44.1%), Fluroquinolones (Ciprofloxacin) for 12 (35.3%), first-generation cephalosporins (Cephalothin, Cefazolin, and Cephalexin) for 4 (11.8%) cases and carbapenems (Meropenem) in one (2.9%) patient. Glycopeptides, aminoglycosides, macrolides, sulfonamides, second and fourthgeneration cephalosporins, penicillin, amoxicillin, co-amoxiclav, and cloxacillin were used in none of the patients.

Among the patients with super-infection, four had fungal infections with aspergillosis, which compromises 11.4% of superinfection, 0.79% of patients, and 0.68% of all operations. *Aspergillus fumigatus* was the causative pathogen in all four patients that were diagnosed with fungal superinfection. The features of these four patients are demonstrated in Table 3. As demonstrated, the male-to-female ratio was 1:1, and all patients were from urban areas. None of the patients had a previous history of CE or any other comorbid disease. All of these cases were in the lung and during the first presentation of the disease. All patients were discharged in relatively well condition.

**Table 3:** Evaluation of features of patients with fungal super-infection of hydatid cyst among surgical cases in Southern Iran from 2004–2018

Factor	Case 1	Case 2	Case 3	Case 4
Age/gender	30 y/o male	36 y/o female	55 y/o female	58 y/o male
Hospitalization duration	12 days	10 days	30 days	18 days
Location of cyst in lung	Both lungs	Lt upper lobe	Bilateral (Rt lower lobe + Upper Lt)	Rt lower lobe superior segment
Diameters (cm)	2 × 1 and 3 × 2	6 × 6	5 × 5 × 4 and 7 × 4 × 2	5.8 × 4.3
Radiological Imaging	Xray: hydatid cyst	Xray + CT: hydatid cyst along with multiple small lesions	Xray + CT: collapsed Lt lung + demonstrating hydatid cyst	Xray + CT: hydatid cyst along with surrounding infiltration
WBC (x10^9^/ml)	7.20	7.27	6.30	11.10
Hemoglobin (g/dL)	13.3	13.5	11.6	9.9
Platelet (x10^3^/ml)	416	259	200	445
Symptoms	Cough and Bronchorrhea (1 day)	Fever, Cough, Sputum, and Hemoptysis	Dyspnea + Cough (2 months)	Cough, Malaise, Fever, Hemoptysis (3 months)
Surgical operation	Evacuation and Resection	Resection	Fiberoptic Bronchoscopy + Lt posterolateral thoracotomy + Resection Rt Upper lobe + Right Lateral Thoracotomy	Rt lower lobectomy, mediastinal lymphadenectomy, and Rt upper lobe apical segmentectomy
Medication	Albendazole, Ceftriaxone, Ciprofloxacin, Clindamycin	Ceftriaxone, Metronidazole, Ciprofloxacin, Clindamycin	Ceftriaxone, Ciprofloxacin, Clindamycin	Albendazole, Metronidazole, Clindamycin
Notes	-	-	Ruptured hydatid cyst + Collapsed Lt lung + Bacterial Empyema	CCIEP Positive

CCIEP: Countercurrent immunoelectrophoresis; CT: Computed tomography; Lt: Left; Rt: Right; WBC: White blood cell count

## Discussion

Superinfection of CE is considered a life-threatening complication with significant morbidities, which can be prevented with early diagnosis and treatment. Our study revealed a 5.99% incident rate of CE superinfection, which falls in the range of previous reports, reporting an approximate incidence of 1–8% for superinfected hydatid cysts [13, 14]. However, almost all previous studies investigated the overall incidence of the disease in hospital settings. Therefore, a potential selection bias should be taken into consideration in terms of an overestimated incidence of superinfection in patients with hydatid cysts.

In the literature, the prevalence of superinfection in hydatid cysts of the liver has typically been higher than in the lungs [7]. However, in our study, we found the opposite, with a higher rate of superinfection in hydatid cysts of the lung. Interestingly, our results indicated a lower likelihood of superinfection in the upper lobes of the lungs. Accordingly, out of the 20 patients with an infected hydatid cyst in lungs, 25% (5 patients) had the infection in the upper lobes. A similar pattern was observed for infected hydatid cysts in the liver, with a higher prevalence of superinfection in the right liver lobe. This difference may be due to the higher overall incidence of CE in the right hepatic lobe, which has been speculated to be due to increased blood flow to that side [15].

Conventionally, it has been thought that CE superinfection occurs almost always as a sequala of cyst rupture [14]. However, only one patient with super-infection in our study had a diagnosis of cystic rupture. Although other factors including bacteremia or distortion of adjacent structures by the enlarged cyst have been introduced as a cause of superinfection, the exact pathophysiology for superinfection remains unclear [7]. According to our study, laboratory tests including eosinophil counts, were not effective at distinguishing between patients with and without superinfection. These routine blood tests showed no clear differences between the two groups of patients. Although, in cases where the diagnosis is uncertain, the presence of eosinophilia may support the diagnosis [16].

While radiographic imaging modalities are reliable for initially diagnosing CE, they may not be efficient for detecting infected cysts. Our study found that there were no discernible differences in the physical characteristics of infected hydatid cysts compared to those that were not infected. Accordingly, the location, size, and presence of necrosis in hydatid cysts were not reliable indicators of superinfection. As previously reported by Manterola et al. [17], it is not possible to accurately distinguish an infected hydatid cyst from a non-complicated one based on the imaging findings alone. Regardless, the gold standard diagnostic approach for an infected CE seems to be the histologic evaluation of the drained materials from the cyst itself.

In terms of fungal superinfection, there are very limited data on the optimal diagnostic and therapeutic approaches. Most reports of superinfected hydatid cysts by fungal pathogens have been described in small case series and case reports [7, 18, 19]. Accordingly, the most robust data on fungal CE superinfection came from the study by Kocer et al., who reported that out of 100 patients with a preliminary diagnosis of CE, only two patients had superinfected hydatid cysts by aspergillosis [9]. Results from the reported cases in the literature and our study point to the fact that the fungal CE superinfection is a rare entity. In our experience, *Aspergillus fumigatus* was the causative fungi in all four patients with fungal superinfection. No patients had a history of immunodeficiencies or any comorbid diseases. Although the presence of immune deficiency may predispose patients to disseminated disease, the majority of reported fungal CE coinfections were immunocompetent patients [7, 11, 18, 20] Therefore, the possibility of superinfection and complicated CE should not be ruled out in the absence of immunodeficiency.

Notably, preoperative serologic and imaging findings were inconclusive for the definite diagnosis. Nevertheless, some studies suggested that serum vascular endothelial growth factor can be used as a potential serum marker for the diagnosis of pulmonary aspergilloma [21]. However, its role in the diagnosis of fungal CE superinfection remains unclear. In our study, only one patient with fungal superinfection had a concomitant CE rupture, that underwent right upper lobe resection due to massive cyst infiltration to the adjacent tissues. Generally, the main treatment option for superinfected CE is surgical resection, which is mostly curative [22]. A systematic review of 22 patients with fungal CE superinfection showed that lobectomy was most commonly used as the preferred surgical method for superinfected pulmonary CE by fungal pathogens [19]. However, the degree of surgical resection mainly depends on the cystic location and size, as well as the extent of local invasion.

In our study, anti-helminth therapy was administered to 15 (44.1%) patients, and two out of four fungal infected cases to prevent a disseminated infection. Fortunately, none of them progressed with systemic fungal infection at any stage of the treatment. The use of anti-helminthic therapy has been a subject of significant debate. According to the study by Aliyali et al. [19], 29% of pulmonary fungal CE superinfections in the literature received anti-helminthic medications, which showed no significant difference in the overall patients’ outcomes. Also, a 28-year experience by Chen et al. [22] showed that despite anti-helminth treatment, 14 (73.7%) patients remained symptomatic and required surgical excision for disease control. Notably, they observed that 60% of patients that did not undergo surgical excision, died due to massive hemoptysis [22]. Overall, anti-helminth therapy is not generally indicated and remains only for immunocompromised patients and patients with severe comorbidities with a considerable risk for disseminated infection [9, 11].

It is worth noting that this study had several limitations. Since our study location is among the endemic locations of hydatid cyst, and based on the high number of patients and operations, the contents are not usually sent for gram stain and culture antibiogram evaluation, since the results of these procedures does not change the method of management in hydatid cyst patients. If a patient developed further complications in favor of infection during their post-operation course, culture sample were sent to evaluate possible causative agents; however, we did not observe any of these cases among our hospital records and all of our subjects had an uneventful post-operative course with no significant complications during follow-ups. Another limitation is the retrospective design of the study; therefore, drawing a precise causative relation between the patients’ features and the incidence of superinfection was not possible. Also, due to the rarity of fungal superinfections, there is no quantitative analysis available and a definite consensus was not achievable. Regardless, this study has one of the most robust data regarding the clinical characteristics and the long-term patient outcomes of superinfected hydatid cysts in the current literature.

## Conclusion

Our study revealed a 5.99% incident rate of CE superinfection. Regarding the concurrent fungal infections in hydatid cysts, the patient’s symptoms and laboratory and imaging findings are inconclusive and histopathological evaluation seems to be the most reliable option. Surgical resection is the gold-standard treatment option with favorable outcomes and potentially can be curative. Our study demonstrated the lung to be the most frequent location of CE superinfection, and *Aspergillus fumigatus* as the most common causative fungi.

## Data Availability

SPSS data of the participant can be requested from the authors. Please write to the corresponding author if you are interested in such data.
